# Magnetic suppression of perceptual accuracy is not reduced in episodic migraine without aura

**DOI:** 10.1186/1129-2377-15-83

**Published:** 2014-12-03

**Authors:** Veronika Rauschel, Ruth Ruscheweyh, Thomas Eggert, Andreas Straube

**Affiliations:** 1 Department of Neurology, University of Munich, Marchioninistr. 15, 81377 Munich, Germany

**Keywords:** Magnetic suppression of perceptual accuracy, Transcranial magnetic stimulation, Migraine, Cortical excitability, Test-retest reliability

## Abstract

**Background:**

Altered cortical excitability is thought to be part of migraine pathophysiology. Reduced magnetic suppression of perceptual accuracy (MSPA) has been found in episodic migraine with aura and in chronic migraine, and has been interpreted as reduced inhibition of the occipital cortex in these migraine subtypes. Results are less clear for episodic migraine without aura. In the present study we compared MSPA between 24 healthy controls and 22 interictally measured episodic migraine patients without aura. In addition, we investigated test-retest reliability in 33 subjects (24 controls, 9 migraine).

**Findings:**

Visual accuracy was assessed by letter recognition and modulated by transcranial magnetic stimulation delivered to the occipital cortex at different intervals to the letter presentation (40, 100 and 190 ms). The results confirm suppression of visual accuracy at the 100 ms interval (p < 0.001), but there were no significant group differences (percentage of correctly recognized letters, control: 36.1 ± 36.2; migraine: 44.0 ± 32.3, p = 0.44). Controls and migraine patients were pooled for assessment of test-retest reliability (n = 33). Levels of suppression at 100 ms were similar at test (percentage of correctly recognized letters: 42.3 ± 32.6) and retest (41.9 ± 33.8, p = 0.90) and test-retest correlations were good (r = 0.82, p < 0.001).

**Conclusions:**

The results demonstrate that occipital cortex inhibition as assessed with MSPA is not reduced in episodic migraine without aura. This suggests a larger role of occipital cortex excitability in episodic migraine with aura and in chronic migraine compared to episodic migraine without aura. Test-retest reliability of MSPA was good.

## Findings

### Background

Migraine is a disabling disorder, affecting roughly 13% of the adult population [1]. Altered cortical excitability likely contributes to migraine pathophysiology [2]. Visual aura is the most prevalent aura symptom in migraine and is clearly related to the excitability of the visual cortex [3]. Therefore, the occipital cortex is believed to play a prominent role in migraine pathophysiology. One way to assess occipital cortex excitability is to measure magnetic suppression of perceptual accuracy (MSPA). In this technique, a single transcranial magnetic pulse (TMS) delivered over the occipital cortex impairs recognition of letters presented on a screen 100 ms before the pulse [4,5]. MSPA has been shown to be reduced in episodic migraine with aura [5,6] and in chronic migraine [7,8], which has been interpreted as a lack of occipital intracortical inhibition. Results are less clear for episodic migraine without aura. One study found no difference in MSPA between controls and subjects with episodic migraine without aura, however, the groups were relatively small (14 migraine patients without aura and 13 controls) and migraine frequency was low (1.5 ± 1.1 migraine attacks/month) [6]. Two studies showed reduced MSPA in episodic migraine (5 episodic migraine patients, 5 controls), but it was not stated if the patients suffered from migraine with or without aura [7,8].

In the present study, we investigated a larger sample of controls (n = 24) and episodic migraine patients without aura (n = 22) that were more severely affected (4.9 ± 2.2 headache days/month) to provide more conclusive evidence if MSPA is reduced in migraine without aura. As cortical excitability has been shown to change around the migraine attack [2], all patients were tested in the interictal phase. In addition, we determined the test-retest reliability of MSPA by a second measurement 2–3 weeks later.

### Methods

#### ***Subjects***

Experiments were performed at the Department of Neurology of the University of Munich. The study was conducted in accordance with the Declaration of Helsinki and approved by the local ethics committee. Subjects provided written informed consent.

25 controls and 25 episodic migraine patients without aura with normal or corrected-to-normal vision were recruited by advertisements on the campus. Migraine, and the absence of other headache types, was diagnosed according to the International Classification of Headache Disorders (ICHD-III) [9] by an experienced physician from the supraregional outpatient headache clinic at the Department of Neurology. Controls had to be free of any headaches. Participants had to be free of migraine preventive medication for at least 4 weeks prior to participation. Analgesic medication and triptans were allowed, but not within 48 hours before the experiment. Migraine patients were tested interictally (no headache within 48 hours before and after the experiment). Subjects were investigated two times within 2–3 weeks. Three migraine patients who developed headache within 48 hours after both sessions and one control who was not able to tolerate TMS had to be excluded, leaving 22 migraine patients (age 28.1 ± 6.9; 1 male) and 24 controls (age 25.3 ± 6.2; 3 males, Table 1) for the group comparison. An additional 13 migraine patients developed headache within 48 hours after one of the two sessions, leaving 9 migraine patients for the test-retest analysis. For group comparison, control subjects were matched to migraine patients for use of data from the first (16 control, 13 migraine) or second (8 control, 9 migraine) session. The high incidence of headache after the sessions may be due to the fact that TMS can induce headaches [10].

**Table 1:** Description of the cohort (mean ± SD)

	**Control**	**Episodic migraine without aura**
**n**	24	22
**Age**	25.3 ± 6.2	28.1 ± 6.9
**Gender (male:female)**	3:21	1:21
**Headache history (years)**	-	10.7 ± 6.1
**Headache days/month**	-	4.9 ± 2.2
**Headache intensity (1–10)**	-	6.8 ± 1.3

#### ***Assessment of MSPA***

MSPA was measured according to published methods [7,8,11,12] and is described in detail in Additional file 1: Methods. Briefly, subjects were seated in front of a computer monitor on which trigrams (3-letter sequences) were flashed for 30 ms. Subjects were asked to report the letters in the correct order. During a training run, the contrast was adjusted so that approximately 80% of the letters were correctly recognized. During the experimental run, presentation of every trigram was followed at randomized intervals of 40, 100 and 190 ms by a TMS pulse of 70% maximal output (Magstim 200, The MagStim Company Ltd, Whitland, UK) delivered via a 90 mm circular coil to the occipital cortex. The interval between the start of the trigram presentation and the delivery of the TMS pulse is called stimulus onset asynchrony (SOA). 54 trials were performed and the percentage of correctly recognized letters was calculated for each SOA interval. During the retest session, the contrast was identical to the first session but a training run was performed nonetheless.

#### ***Statistical analysis***

Data was analyzed using the Statistical Package for Social Sciences (SPSS, version 22, IBM Corporation, Armonk, New York, USA). Values are given as mean ± standard deviation (SD) unless otherwise stated. P < 0.05 was considered significant. Age and sex were compared between groups using t-test and chi-square test respectively. MSPA was compared between groups using ANOVA (within-subject factor: SOA, between-subject factor: group), t-tests and Bonferroni posthoc tests as appropriate. Test-retest reliability was assessed using ANOVA (within-subject factors: session and SOA), t-tests, and Pearson’s and intraclass correlation coefficients (ICC) as appropriate.

### Results

Characteristics of the study cohort are given in Table 1. Age and sex were not significantly different between controls and migraine patients (age: T[44] = 1.5, p = 0.15, sex: χ^2^[1] =0.9, p = 0.34).

**Figure 1:**
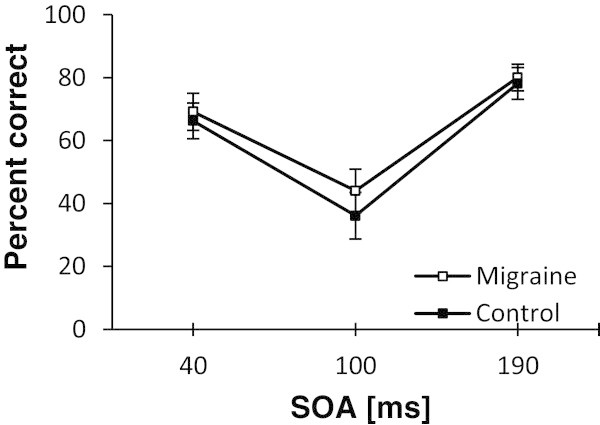
**Group differences in magnetic suppression of perceptual accuracy (MSPA).** MSPA profiles are illustrated for interictal migraine patients (n = 22) and controls (n = 24) as mean ± SEM. SOA, stimulus onset asynchrony (time in milliseconds between the appearance of the trigram and the delivery of TMS). The y-axis illustrates the percentage of correctly identified letters.

MSPA profiles of controls (n = 24) and migraine patients (n = 22) are shown in Figure 1 and Additional file 1: Table S1. A significant main effect of SOA interval (F[1.6] = 58.1, p < 0.001), and posthoc tests (all p < 0.001) confirmed suppression of visual accuracy at SOA intervals of 100 ms, compared to 40 or 190 ms. There was no significant interaction between SOA interval and group (F[1.6] = 0.4, p = 0.64) and no significant difference in the percentage of correctly recognized letters at 100 ms SOA between migraine and control subjects (T[44] = 0.78, p = 0.44). In addition, there was no correlation between the percentage of correctly recognized letters at 100 ms SOA and the number of headache days per month in migraine patients (r = -0.13, p =0.56).

**Figure 2:**
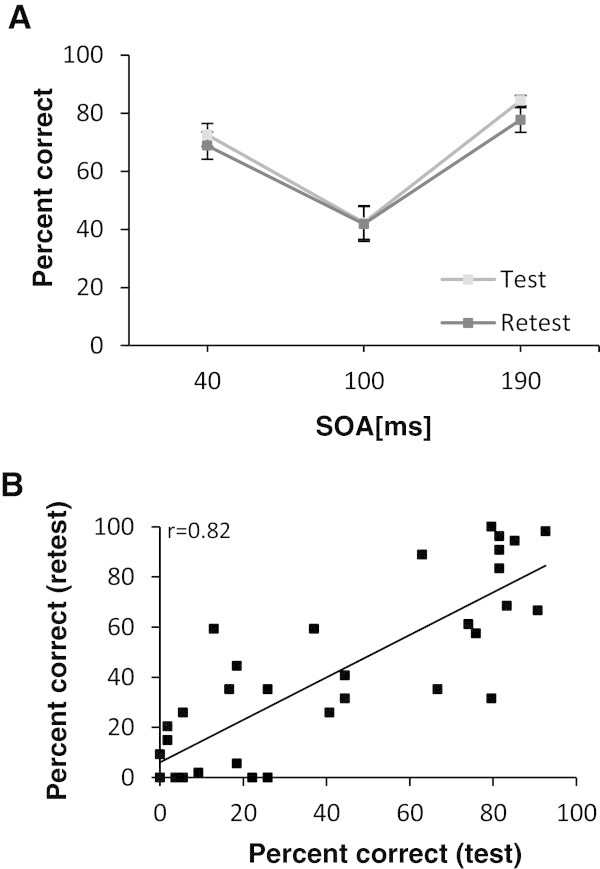
**Test-retest reliability of magnetic suppression of perceptual accuracy (MSPA). A.** MSPA profiles (percentages of correctly identified letters) are illustrated for 33 subjects (22 controls, 9 migraine patients) at baseline and at retest 2–3 weeks later (mean ± SEM). **B.** Correlations between correctly identified letters at 100 ms SOA at baseline and at retest (2–3 weeks later) are illustrated. The linear regression line and corresponding Pearson’s r are given. SOA, stimulus onset asynchrony (time in milliseconds between the appearance of the trigram and the delivery of TMS).

Test-retest reliability over 2–3 weeks was assessed in 24 controls and 9 migraine patients and is shown in Figure 2 and Additional file 1: Table S2. Because of the low number of migraine patients with two interictal measurements available, groups were pooled for assessment of test-retest reliability. ANOVA revealed no main effect of session (F[1,32] = 1.81, p = 0.19) and no interaction between SOA interval and session (F[1.9] = 1.1, p = 0.33). Also, there was no significant difference in the percentage of correctly recognized letters at 100 ms SOA between migraine and control subjects (T[32] = 0.13, p = 0.90). Correlations of percent correctly recognized letters at 100 ms SOA between test and retest were high (r = 0.82, ICC = 0.82, p < 0.001). It has been proposed that because of different rates of basal recognition of letters, a suppression score (percentage of correctly recognized letters at 100 ms SOA divided by maximum percentage of correctly recognized letters at any other SOA) would be a more reliable measure of MSPA [7,8]. Results using suppression scores were equivalent to those shown above (see Additional file 1: Results).

### Discussion

The main result of the present study is that MSPA is not reduced in episodic migraine without aura. This confirms data from a previous study that showed reduced MSPA in migraine with aura, but not in migraine without aura in a smaller and less severely affected cohort (14 patients with migraine without aura, 1.5 ± 1.1 migraine attacks/month, [7]). It has been suggested that MSPA is the result of direct activation of inhibitory interneurons in the occipital cortex by the TMS pulse, and that reduced MSPA indicates impairment of occipital inhibitory neural networks, resulting in functional hyperexcitability [13,6]. Occipital hyperexcitability may favour cortical spreading depression, which is thought to initiate migraine attacks with visual aura [14]. In contrast, occipital spreading depression does not seem to play a major role in migraine without aura [15]. Reduced MSPA in migraine with aura but not in migraine without aura is consistent with these pathophysiological ideas. However, previous studies have also shown reduced MSPA in chronic migraine [7,8], which in most cases develops from migraine without aura. The present results, with no correlation between MSPA and number of headache days, do not directly support the concept of MSPA decreasing with migraine frequency, reaching minimal values in chronic migraine [7,8]. A large proportion of the chronic migraine patients tested previously overused acute headache medication [7,8]. In addition, it is difficult or even impossible to test chronic migraine patients interictally. This is important since cortical excitability changes rapidly in the peri-ictal period [16]. Maybe medication overuse and/or peri-ictal testing may have contributed to the MSPA reduction reported in chronic migraine [7,8]. Further studies will be necessary to clarify this point.

A previous study has shown good reproducibility of MSPA group mean values at test and retest after 2 weeks in 10 healthy volunteers [12]. Our results confirm these results in a larger group (n = 33) also including migraine patients and complete these findings by also assessing the test-retest correlation, which was good. Unfortunately, only 9 migraine patients could be tested interictally during test and retest, precluding a separate analysis of test-retest reliability in controls and migraine patients.

## Abbreviations

MSPA: Magnetic suppression of perceptual accuracy; TMS: Transcranial magnetic stimulation; ICHD: International classification of headache disorders; SOA: Stimulus onset asynchrony; SPSS: Statistical Package for Social Sciences; SD: Standard deviation; ANOVA: Analysis of variance; ICC: Intraclass correlation coefficient.

## Competing interests

The authors declare that they have no competing interests.

## Authors’ contributions

VR participated in conceptualizing and designing the study, performed the experiments and the data analysis, drafted the initial manuscript and approved the final version of the manuscript. TE participated in the study design, programmed the MSPA paradigm, revised the manuscript and gave approval of the final version of the manuscript. RR participated in conceptualizing and designing the study, supervised the experiments and the data analysis, revised the manuscript and gave approval of the final version of the manuscript. AS participated in conceptualizing and designing the study, revised the manuscript and gave approval of the final version of the manuscript. All authors read and approved the final manuscript.

## Supplementary Material

Additional file 1 **Methods.** Apparatus and procedures in detail. **Results.** Analysis of suppression scores. **Table S1.** MSPA group comparison. **Table S2.** MSPA profiles at baseline and retest. Click here for file

## References

[B1] Lipton R Scher A Kolodner K Liberman J Steiner SW Migraine in the United States: epidemiology and patterns of health care use Neurology 2002 58 6 885 894 10.1212/WNL.58.6.885 11914403

[B2] Coppola G Pierelli F Schoenen J Habituation and migraine Neurobiol Learn Mem 2009 92 2 249 259 10.1016/j.nlm.2008.07.006 18675928

[B3] Purdy R The role of the visual system in migraine: an update Neurol Sci 2011 32 Suppl 1 S89 S93 21533721 10.1007/s10072-011-0541-4

[B4] Amassian V Cracco R Maccabee P Cracco J Rudell A Eberle L Suppression of visual perception by magnetic coil stimulation of human occipital cortex Electroencephalogr Clin Neurophysiol 1989 74 6 458 462 10.1016/0168-5597(89)90036-1 2480226

[B5] Mulleners W Chronicle E Palmer J Koehler P Vredeveld J Suppression of perception in migraine: evidence for reduced inhibition in the visual cortex Neurology 2001 56 2 178 183 10.1212/WNL.56.2.178 11160952

[B6] Chronicle E Pearson A Mulleners W Objective assessment of cortical excitability in migraine with and without aura Cephalalgia 2006 26 7 801 808 10.1111/j.1468-2982.2006.01144.x 16776694

[B7] Aurora S Barrodale P Chronicle E Mulleners W Cortical inhibition is reduced in chronic and episodic migraine and demonstrates a spectrum of illness Headache 2005 45 5 546 552 10.1111/j.1526-4610.2005.05108.x 15953273

[B8] Aurora S Barrodale P Tipton R Khodavirdi A Brainstem dysfunction in chronic migraine as evidenced by neurophysiological and positron emission tomography studies Headache 2007 47 7 996 1003 discussion 1004–7 10.1111/j.1526-4610.2007.00853.x 17635590

[B9] Headache Classification Committee of the International Headache Society (IHS) The international classification of headache disorders, 3rd edition (beta version) Cephalalgia Int J Headache 2013 33 9 629 808 10.1177/0333102413485658 23771276

[B10] Maizey L Allen C Dervinis M Verbruggen F Varnava A Kozlov M Adams R Stokes M Klemen J Bungert A Hounsell C Chambers C Comparative incidence rates of mild adverse effects to transcranial magnetic stimulation Clin Neurophysiol 2013 124 3 536 544 10.1016/j.clinph.2012.07.024 22986284

[B11] Aurora S Barrodale P Vermaas A Rudra C Topiramate modulates excitability of the occipital cortex when measured by transcranial magnetic stimulation Cephalalgia Int J Headache 2010 30 6 648 654 10.1111/j.1468-2982.2009.01998.x 19732073

[B12] Custers A Mulleners W Chronicle E Assessing cortical excitability in migraine: reliability of magnetic suppression of perceptual accuracy technique over time Headache 2005 45 9 1202 1207 10.1111/j.1526-4610.2005.00243.x 16178950

[B13] Moliadze V Zhao Y Eysel U Funke K Effect of transcranial magnetic stimulation on single-unit activity in the cat primary visual cortex J Physiol 2003 553 Pt 2 665 679 12963791 10.1113/jphysiol.2003.050153 PMC2343567

[B14] Charles A Baca S Cortical spreading depression and migraine Nat Rev Neurol 2013 9 11 637 644 10.1038/nrneurol.2013.192 24042483

[B15] Moskowitz M Genes, proteases, cortical spreading depression and migraine: impact on pathophysiology and treatment Funct Neurol 2007 22 3 133 136 17925161

[B16] Cosentino G Fierro B Vigneri S Talamanca S Paladino P Baschi R Indovino S Maccora S Valentino F Fileccia E Giglia G Brighina F Cyclical changes of cortical excitability and metaplasticity in migraine: evidence from a repetitive transcranial magnetic stimulation study Pain 2014 155 6 1070 1078 10.1016/j.pain.2014.02.024 24631596

