# Supplementary material for: Magnetic suppression of perceptual accuracy is not reduced in episodic migraine without aura
Source: J Headache Pain. 2014 Dec 3;15(1):83. doi: 10.1186/1129-2377-15-83 (PMC4273691; doi:10.1186/1129-2377-15-83)
Supplement: Additional file 1 — Methods. Apparatus and procedures in detail. Results. Analysis of suppression scores. Table S1. MSPA group comparison. Table S2. MSPA profiles at baseline and retest. [file 1129-2377-15-83-S1.docx]

**Magnetic suppression of perceptual accuracy is not reduced in episodic migraine without aura**

**Additional file 1: Methods. Apparatus and procedures in detail**

**Additional file 1: Results. Analysis of suppression scores**

**Additional file 1: Table S1. MSPA group comparison**

**Additional file 1: Table S2. MSPA profiles at baseline and retest**

**Methods. Apparatus and procedures in detail**

Subjects were tested in a darkened room. They were asked to sit in a relaxed but straight position and fixate a frame displayed on a computer monitor (View Sonic Professional Series PT775 monitor, London, Great Britain) at a distance of 100 cm. Trigrams (3 letter sequences) were flashed within the frame for 30 ms duration, subtending a visual angle of 1.73°x0.79°. The letters were chosen from a subset of letters of approximately equal legibility [1] and presented in Arial. Visual stimuli were generated using custom-made software running on a Dell Inc. Optiplex 790 desktop computer (Round Rock, Texas, USA), using Matlab R2011b (The Mathworks Inc, Natick, MA, USA). Subjects were asked to report the letters in correct order and to say “blank” or “don’t know” if they did not recognize a specific letter. Subjects’ letter reports were recorded by the experimenter.

During a training run, blocks of 10 trigrams were shown with ≥5 seconds between trigrams, and contrast of the trigrams was adapted until about 80% of the letters were recognized correctly. Usually 2-3 training blocks were performed.

Occipital monophasic transcranial magnetic stimulation was generated using a MagStim 200 (The MagStim Company Ltd, Whitland, UK) and a 90-mm circular coil, resulting in a maximum output of 2T. A nonfocal coil has been used in all previous studies investigating MSPA in migraine [2,3,4,5] and stimulation with a focal coil has been reported to be less effective for eliciting MSPA [6]. The coil was centered over the midline, lower edge 1 cm above the inion, handle upwards, side B oriented towards the head. To keep the coil in place during the experiment, a coil holder attached to a rack was used.

During the experimental session, presentation of trigrams was followed at randomized intervals of 40, 100 and 190 ms by a single TMS pulse of 70% maximal output. The interval between start of the trigram presentation and delivery of the TMS pulse is called stimulus onset asynchrony (SOA). Previous studies have used a larger number of different SOAs, but have demonstrated maximum suppression of visual accuracy at 100 ms and minimum suppression at 40 and 190 ms [2, 7]. Therefore we limited investigation to these three SOAs, with the advantage that a larger number of trials per SOA could be performed. Trigram presentations were spaced ≥5 seconds. 54 trials were performed and percent of correctly recognized letters was calculated for each SOA interval. 54 different trigrams were presented during the 54 trials. Each of the three SOAs was used 18 times, and randomization of SOAs over the 54 trials was achieved using a free web-based random number generator (GraphPad QuickCalcs, <http://graphpad.com/quickcalcs/randomN1.cfm>). Identical random sequences were used for every subject, but different random sequences were used for training runs and experimental runs and for test and retest.

**Results. Analysis of suppression scores**

It has been proposed that because of different rates of basal recognition of letters, a suppression score (percent of correctly recognized letters at 100 ms SOA divided by maximum percent of correctly recognized letters at any other SOA) would be a more reliable measure of MSPA [2, 3]. Therefore, we repeated the analysis using suppression scores. Suppression scores were 0.40±0.38 for controls and 0.51±0.37 for migraine patients, again without significant group differences (T[44]=1.0 p=0.31). In addition, there was no correlation between MSPA suppression scores and number of headache days per month in migraine patients (r = -0.15, p =0.50).

The test-retest analysis was also repeated with suppression scores. There were no significant differences in mean values of suppression scores (test: 0.53±0.38, retest: 0.50±0.35, T[32]=0.63, p=0.54, n=33) and correlations between test and retest were high (r=0.82, ICC=0.82, both p<0.001).

**References**

1. Grimm W, Rassow B, Wesemann W, Saur K, Hilz R (1994) Correlation of optotypes with the Landolt ring--a fresh look at the comparability of optotypes. Optom Vis Sci 71(1):6-13
2. Chronicle E, Pearson A, Mulleners W (2006) Objective assessment of cortical excitability in migraine with and without aura. Cephalalgia 26(7):801-8
3. Aurora S, Barrodale P, Chronicle E, Mulleners W (2005) Cortical inhibition is reduced in chronic and episodic migraine and demonstrates a spectrum of illness. Headache 45(5):546-52
4. Aurora S, Barrodale P, Tipton R, Khodavirdi A (2007) Brainstem dysfunction in chronic migraine as evidenced by neurophysiological and positron emission tomography studies. Headache 47(7): 996-1003; discussion 1004-7
5. Aurora S, Barrodale P, Vermaas A, Rudra C (2010) Topiramate modulates excitability of the occipital cortex when measured by transcranial magnetic stimulation. Cephalalgia Int J Headache 30(6):648-54
6. Amassian V, Cracco R, Maccabee P, Cracco J, Rudell A, Eberle L (1989) Suppression of visual perception by magnetic coil stimulation of human occipital cortex. Electroencephalogr Clin Neurophysiol 74(6):458-62
7. Mulleners W, Chronicle E, Palmer J, Koehler P,Vredeveld J (2001) Suppression of perception in migraine: evidence for reduced inhibition in the visual cortex. Neurology 56(2):178-83

**Table S1. MSPA group comparison**

|  |  | **Stimulus onset asynchrony (ms)** | | |
| --- | --- | --- | --- | --- |
| **Group** | **n** | **40** | **100** | **190** |
| **Episodic migraine** | 22 | 69.2±27.1 | 44.0±31.5 | 80.1±19.2 |
| **Control** | 24 | 66.3±27.1 | 36.1±35.4 | 78.2±24.2 |
| **Total** | 46 | 67.7±27.4 | 39.9±34.2 | 79.1±22.2 |

Group comparison of magnetic suppression of perceptual accuracy (MSPA) profiles (mean ± SD). Stimulus onset asynchrony (SOA): time in milliseconds between the appearance of the trigram and the delivery of TMS.

**Table S2. MSPA profiles at baseline and retest**

|  |  | **Test** | | | **Retest** | | |
| --- | --- | --- | --- | --- | --- | --- | --- |
|  |  | **Stimulus onset asynchrony (ms)** | | | | | |
| **Group** | **n** | **40** | **100** | **190** | **40** | **100** | **190** |
| **Episodic migraine** | 9 | 78.6±12.0 | 53.1±29.2 | 83.1±9.5 | 79.4±18.8 | 57.4±19.1 | 83.1±10.7 |
| **Control** | 24 | 70.2±24.6 | 38.3±32.8 | 84.7±10.4 | 64.8±27.7 | 36.0±36.2 | 75.6±27.0 |
| **Total** | 33 | 72.5±22.2 | 42.3±32.6 | 84.3±10.2 | 68.8±26.4 | 41.9±33.8 | 77.7±23.9 |

Magnetic suppression of perceptual accuracy (MSPA) profiles at baseline and retest (2-3 weeks later, mean ± SD). Two interictal measurements could be obtained in only 9 of the migraine patients. Stimulus onset asynchrony (SOA): time in milliseconds between the appearance of the trigram and the delivery of TMS.
